# Discovery of Aloperine as a Potential Antineoplastic Agent for Cholangiocarcinoma Harboring Mutant IDH1

**DOI:** 10.3390/ijms25179226

**Published:** 2024-08-25

**Authors:** Xingkang Wu, Yang Li, Chenchen Han, Shifei Li, Xuemei Qin

**Affiliations:** 1 Modern Research Center for Traditional Chinese Medicine, Key Laboratory of Chemical Biology and Molecular Engineering of Ministry of Education, Shanxi University, No. 92, Wucheng Road, Taiyuan 030006, China; ly20171310418@163.com (Y.L.); 15735649396@163.com (C.H.); 2 Key Laboratory of Chemical Biology and Molecular Engineering of Education Ministry, Institute of Molecular Science, Shanxi University, No. 92, Wucheng Road, Taiyuan 030006, China; lisf@sxu.edu.cn

**Keywords:** quinolizidine alkaloids, aloperine, intrahepatic cholangiocarcinoma, D-2-hydroxyglutarate, IDH mutation

## Abstract

Intrahepatic cholangiocarcinoma (ICC) is a universally lethal malignancy with increasing incidence. However, ICC patients receive limited benefits from current drugs; therefore, we must urgently explore new drugs for treating ICC. Quinolizidine alkaloids, as essential active ingredients extracted from *Sophora alopecuroides Linn*, can suppress cancer cell growth via numerous mechanisms and have therapeutic effects on liver-related diseases. However, the impact of quinolizidine alkaloids on intrahepatic cholangiocarcinoma has not been fully studied. In this article, the in vitro anti-ICC activities of six natural quinolizidine alkaloids were explored. Aloperine was the most potent antitumor compound among the tested quinolizidine alkaloids, and it preferentially inhibited RBE cells rather than HCCC-9810 cells. Mechanistically, aloperine can potentially decrease glutamate content by inhibiting the hydrolysis of glutamine, reducing D-2-hydroxyglutarate levels and, consequently, leading to preferential growth inhibition in isocitrate dehydrogenase (IDH)-mutant ICC cells. In addition, aloperine preferentially resensitizes RBE cells to 5-fluorouracil, AGI-5198 and olaparib. This article demonstrates that aloperine shows preferential antitumor effects in intrahepatic cholangiocarcinoma cells harboring the mutant IDH1 by decreasing D-2-hydroxyglutarate, suggesting that aloperine could be used as a lead compound or adjuvant chemotherapy drug to treat ICC harboring the mutant IDH.

## 1. Introduction

ICC, the second most common primary liver cancer, is an almost universally lethal malignancy, the incidence of which has increased by more than 140% in the past four decades [1]. Only 20–30% of patients are suitable for surgical resection, which is the main ICC treatment strategy [1]. However, 50% of patients who undergo surgical resection typically have an R0 resection [2]. For 70–80% of patients, surgical resection is not viable, so systemic therapy is the best option to delay disease progression, but survival remains limited to approximately 1 year [3]. For the past decade, gemcitabine/cisplatin has been considered the most effective first-line treatment regimen [3]. Targeted therapies, including molecules targeting the fibroblast growth factor receptor (FGFR) and IDH, have received FDA approval for use as second-line treatments in approximately 40% of patients harboring these actionable genomic alterations [4]. However, acquired resistance to FGFR and IDH inhibitors is an emerging challenge [5,6]. Therefore, the development of drugs for treating intrahepatic cholangiocarcinoma is in its infancy.

IDH is a key enzyme in cell metabolism, catalyzing isocitrate to produce α-ketoglutarate [7]. There are three kinds of IDH in mammals: IDH1, IDH2, and IDH3. The mutation of IDH1/2 occurs in almost all types of tumors [7,8]. Tumor-associated IDH1/2 mutations are concentrated in arginine residues at their active sites [7]. The incidence of IDH1/2 mutations in ICC is approximately 25%, and the sites with higher mutation frequency are IDH1 R132C/H/L/S, IDH2 R140Q and IDH2 R172K/W [9,10]. The mutation of IDH1/2’s active site gives the enzyme a new function, which catalyzes the conversion of α-ketoglutarate to D-2-hydroxyglutarate (D-2HG) [7]. D-2HG is a carcinogenic metabolite that induces tumors by inhibiting cell death and tumor immunity, thus inducing cell resistance and activating cell proliferation signals [11,12,13,14,15,16]. The concentration of D-2HG in IDH1/2 mutant ICC tumor tissues is typically about 250 times higher than that in IDH1/2 non-mutant ICC tumor tissues [17]. The average concentration of D-2HG in the blood of IDH1/2 mutant ICC patients is about 13 times higher than that of IDH1/2 non-mutant ICC patients [18]. Therefore, the IDH1/2 mutation has become a new diagnostic and therapeutic marker for ICC. Exploring targeted drugs for treating IDH1/2 mutant ICC is a research hotspot in ICC treatment.

Natural products are an important source of drugs or lead compounds used for the treatment of major diseases and are indispensable in drug development, exemplified by the numerous approved anticancer drugs that prominently feature the scaffolds of their constituents [19]. It has been reported that quinolizidine alkaloids, as essential active ingredients extracted from *Sophora alopecuroides Linn*, exhibit low toxicity and high water solubility, thus having various potential pharmacological applications via numerous mechanisms [20,21,22,23,24,25]. Compound Kushen Injection is a traditional Chinese medicine formula that contains abundant quinolizidine alkaloids, and it has been used to treat liver cancer clinically for the last 20 years [26,27]. Matrine has shown antitumor effects in cholangiocarcinoma and gallbladder cancer cells by suppressing cell proliferation, invasion, and migration by regulating the JAK2/STAT3 and PI3K/AKT signaling pathways [28,29,30]. Moreover, matrine-type quinolizidine alkaloids, matrine, and oxymatrine, have been approved for the clinical treatment of hepatitis B in China [31]. The sophoridine α-aryl propionamide derivative ZM600 has a protective effect on liver fibrosis, thus providing a new candidate for the treatment of liver fibrosis [32]. Thus, quinolizidine alkaloids are key lead compounds for the treatment of hepatobiliary diseases. However, the impacts of quinolizidine alkaloids on ICC have not been fully studied.

**Figure 1:**
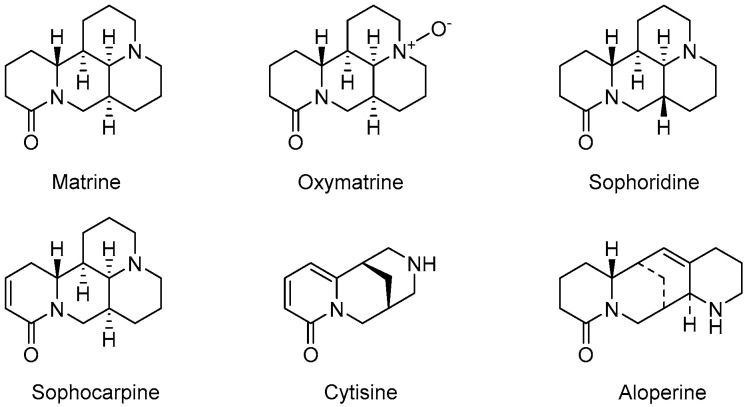
The structures of six quinolizidine alkaloids.

To explore the effects of quinolizidine alkaloids on ICC, we first used two ICC cell lines (RBE and HCCC-9810 cells) to perform in vitro antitumor assays of six well-known quinolizidine alkaloids, namely, matrine, oxymatrine, sophoridine, sophocarpine, cytisine, and aloperine (Figure 1). Fortuitously, all six quinolizidine alkaloids showed certain in vitro anti-ICC activity with selective inhibitory effects in RBE and HCCC-9810 cells. Aloperine was found to be the most potent antitumor compound against ICC cells among these six quinolizidine alkaloids. To further understand the mechanisms underlying the anti-ICC activities of aloperine, we conducted metabonomic and molecular biological experiments, which revealed that aloperine had preferential antitumor effects in ICC cells harboring the mutant IDH1 by decreasing D-2HG. In addition, aloperine preferentially resensitized RBE cells to 5-fluorouracil, AGI-5198, and olaparib. Our results suggest that aloperine could be developed as a lead compound or adjuvant chemotherapy drug to treat ICC harboring the mutant IDH, which is crucial for its further development and utilization.

## 2. Results

### 2.1. The Antitumor Activities of Quinolizidine Alkaloids against ICC Cells

**Figure 2:**
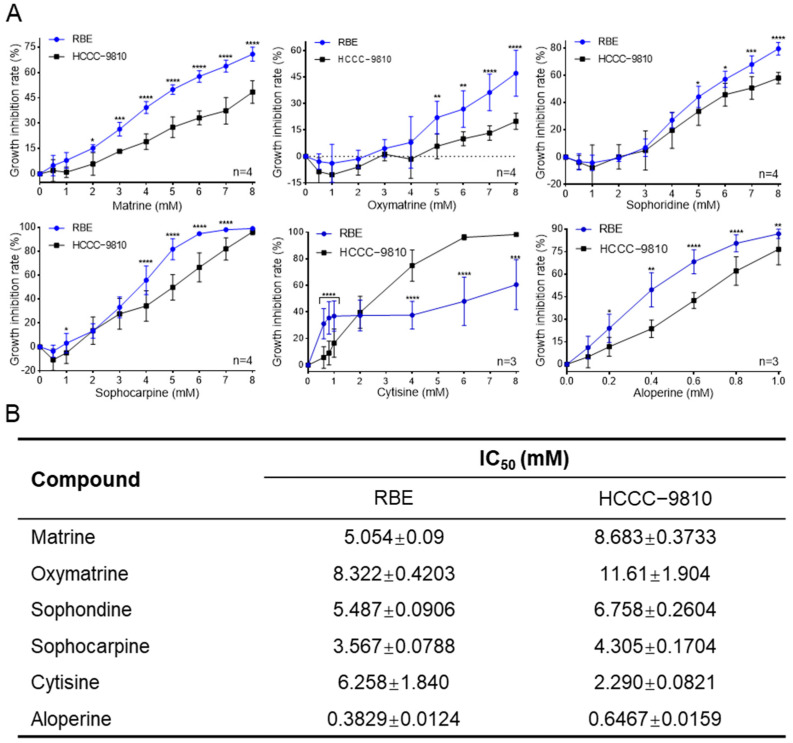
Aloperine was the most active anti-ICC compound among the six quinolizidine alkaloids. (**A**) The cell growth inhibitory activities of the six quinolizidine alkaloids against ICC cells. Two ICC cell lines, including RBE and HCCC-9810 cells, were treated by increasing concentrations of indicated compounds for 72 h, before being subjected to in vitro antitumor assays. The data are presented as the mean ± SEM (*n* ≥ 3). * *p* < 0.05, ** *p* < 0.01, *** *p* < 0.001, and **** *p* < 0.0001. (**B**) The IC_50_ values of six quinolizidine alkaloids against ICC cells. The IC_50_ values were calculated using GraphPad Prism 8 software in nonlinear regression analysis mode.

RBE and HCCC-9810 cells were treated with six quinolizidine alkaloids, namely, matrine, oxymatrine, sophoridine, sophocarpine, cytisine, and aloperine, for 72 h. All of the tested compounds exhibited in vitro antitumor activities in a dose-dependent manner, but sensitivity to the six compounds differed considerably between the two ICC cell lines tested (Figure 2A). Matrine, oxymatrine, sophoridine, sophocarpine, and cytisine had relatively small effects on the cell viability of the two ICC cell lines, with the IC_50_ values being approximately 2–12 mM (Figure 2B). In contrast, the IC_50_ values of aloperine against RBE and HCCC-9810 cells were about 0.3829 mM and 0.6467 mM, tenfold more potent than those of the other five alkaloids (Figure 2B). Strikingly, RBE cells displayed higher sensitivity to the six compounds than HCCC-9810 cells, while four commonly used antitumor drugs, namely, 5-fluorouracil, cisplatin, etoposide, and doxorubicin, showed similar inhibitory activity between RBE and HCCC-9810 cells (Figure 2B and Figure S1). Thus, aloperine is the most potent antitumor compound among the tested quinolizidine alkaloids, having selective inhibitory effects in RBE and HCCC-9810 cells.

### 2.2. Metabolomics Analysis of ICC Cells Treated with Aloperine

**Figure 3:**
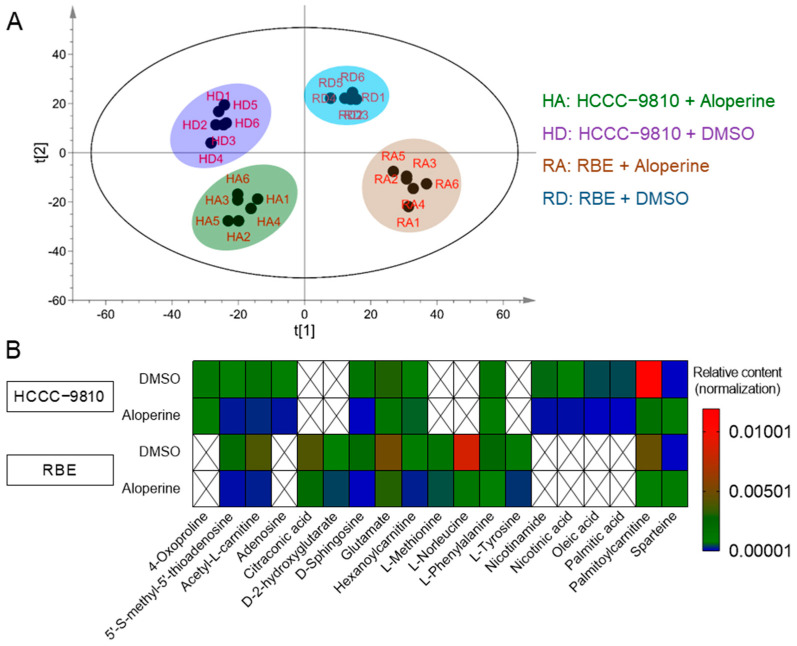
The effects of aloperine on the metabolism of RBE and HCCC-9810 cells. (**A**) An overview of the effects of aloperine on the metabolic profiles of ICC cells. RBE and HCCC-9810 cells were treated with aloperine (0.4 mM) for 24 h before being subjected to untargeted metabolomics analysis. Then, the metabolic profiles of 24 independent samples were determined via PCA analysis. (**B**) The levels of differential metabolites in RBE and HCCC-9810 cells treated with aloperine. The differential metabolites were obtained via OPLS-DA analysis and identified using Compound Discoverer 3.0 software.

To explore the mechanism through which aloperine influenced selective antitumor activity in RBE and HCCC-9810 cells, untargeted metabolomics was used to investigate the effect of aloperine on both cells’ metabolisms. PCA analysis was applied to analyze the metabolic profiles of 24 samples, which were prepared from RBE and HCCC-9810 cells treated with 0.4 mM of aloperine. The 24 samples were clustered into four categories, consistent with the drug treatment group (Figure 3A). The aloperine-untreated groups from RBE and HCCC-9810 cells were evidently separated along the t[1] axis (Figure 3A), indicating that RBE and HCCC-9810 cells possessed different metabolic characteristics, which may be the reason for the cells’ different degrees of sensitivity to aloperine. Meanwhile, aloperine-treated and aloperine-untreated groups were separated along the t[2] axis (Figure 3A). In particular, the regulation of aloperine on metabolites among two cell lines showed a similar tendency (Figure 3A). Thus, aloperine reprogramed the metabolic profiles of RBE and HCCC-9810 cells, revealing that cellular metabolites were potentially related to the antitumor action of aloperine in ICC cells.

Then, OPLS-DA analysis was performed to screen the critical metabolites that were responsible for the differential metabolic profiles of the aloperine-treated experimental group and untreated control group (Figure S2). The permutation test performed via OPLS-DA analysis showed that R2 values were larger than Q2 values, and the regression lines of R2 and Q2 intersected on the right, suggesting that the OPLS-DA analysis model was effective (Figure S2). Meanwhile, S-plots of OPLS-DA analysis displayed differential metabolites with varying importance in terms of projection (VIP > 1) and *p*-values (*p* < 0.05) (Figure S2). Then, by identifying the names of differential metabolites, 19 differential metabolites were obtained (Figure 3B). In both RBE and HCCC-9810 cells, aloperine decreased the levels of 5’-S-methyl-5’-thioadenosine, acetyl-L-carnitine, D-sphingosine, glutamate, hexanoylcarnitine, and palmitoylcarnitine (Figure 3B). Aloperine reduced the levels of citraconic acid, D-2-hydroxyglutarate (D-2HG), L-methionine, L-norleucine, and L-tyrosine in RBE cells, whereas it reduced the levels of adenosine, nicotinamide, nicotinic acid, oleic acid, and palmitic acid in HCCC-9810 cells (Figure 3B). Overall, aloperine had different metabolite regulatory effects on RBE and HCCC-9810 cells, indicating that the regulation of metabolic pathways determines the cytotoxicity sensitivity of aloperine.

### 2.3. D-2HG Is Involved in the Antitumor Activity of Aloperine against RBE Cells

**Figure 4:**
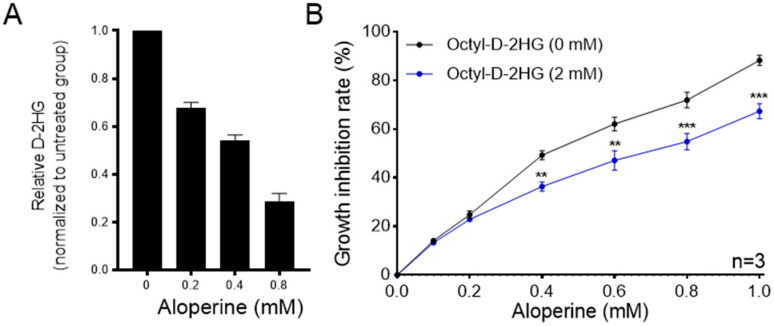
Aloperine preferentially inhibited RBE cells by decreasing glutamate D-2HG (D-2HG) levels. (**A**) Aloperine decreased the levels of D-2HG in RBE cells. RBE cells were treated by increasing concentrations of aloperine for 24 h and subjected to LC–MS quantification analysis. The data are presented as the mean ± SEM (*n* = 3). (**B**) Exogenous D-2HG supplementation reduced the antitumor activity of aloperine against RBE cells. RBE cells were co-treated with aloperine and octyl-D-2HG for 72 h and subjected to an in vitro antitumor assay. The data are presented as the mean ± SEM (*n* = 3). ** *p* < 0.01, *** *p* < 0.001.

Surprisingly, the results of untargeted metabolomics showed that aloperine specifically reduced the levels of the oncometabolite D-2HG in RBE cells. To verify the results of untargeted metabolomics, the level of D-2HG in RBE cells was detected via standard compound-based LC–MS quantitative analysis. The results showed that aloperine decreased the levels of D-2HG in a dose-dependent manner (Figure 4A). To explore whether D-2HG is involved in the antitumor action of aloperine against RBE cells, octyl-D-2HG, a membrane-permeant precursor form of D-2HG, was applied to increase intracellular levels of D-2HG. The results showed that exogenous D-2HG supplementation reduced the antitumor activity of aloperine against RBE cells, revealing that D-2HG was involved in the antitumor activity of aloperine against RBE cells.

### 2.4. IDH1/2 Mutation Sensitizes Cells to Aloperine

**Figure 5:**
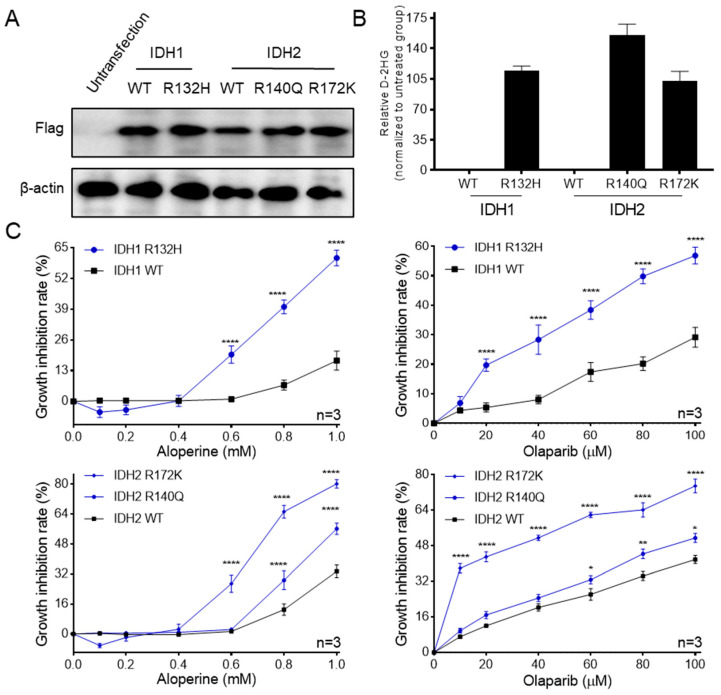
IDH1/2 mutation sensitizes HCCC-9810 cells to aloperine. (**A**) The protein expression of transfected IDH1/2 in HCCC-9810 cells. HCCC-9810 cells, stably expressing wild-type or mutant IDH1/2, were subjected to Western blotting. (**B**) The D-2HG levels of transfected IDH1/2 in HCCC-9810 cells. LC–MS analysis was performed to measure the levels of cellular D-2HG using metabolite standards. The data were presented as the mean ± SEM (*n* = 3). (**C**) The cell growth inhibitory activities of aloperine against HCCC-9810 cells with wild-type or mutant IDH1/2. HCCC-9810 cells, stably expressing wild-type or mutant IDH1/2, were treated by the indicated concentrations of aloperine for 72 h and subjected to in vitro antitumor assays. The data are presented as the mean ± SEM (*n* = 3). * *p* < 0.05, ** *p* < 0.01, and **** *p* < 0.0001.

Given that RBE cells harbor an IDH1 mutation that produces D-2HG [9], while HCCC-9810 cells do not do so, we proposed that the IDH1 mutation affects sensitivity to aloperine in RBE and HCCC-9810 cells. We first treated RBE and HCCC-9810 cells with olaparib and AGI-5198, which are agents targeting IDH-mutant cells. The results showed that the inhibitory activities of olaparib and AGI-5198 on the growth of RBE cells were greater than those on HCCC-9810 cells, indicating that RBE and HCCC-9810 cells, in this study, harbor different IDH mutation statuses (Figure S3). To confirm whether IDH1 mutation affects sensitivity to aloperine in ICC cells, IDH1/2-mutant HCCC-9810 cells were established by stably expressing flag-tagged wild-type IDH1/2 or mutant IDH1/2. Western blotting showed that flag-tagged wild-type IDH1/2 or mutant IDH1/2 was overexpressed in HCCC-9810 cells (Figure 5A). The quantitative analysis of D-2HG showed that overexpressed flag-tagged mutant IDH1/2 increased the levels of D-2HG in HCCC-9810 cells (Figure 5B). These results indicated that IDH1/2-mutant HCCC-9810 cells were successfully identified. Then, IDH1/2-mutant HCCC-9810 cells were subjected to an in vitro antitumor assay. Both IDH1 and IDH2 mutation enhanced the sensitivity of HCCC-9810 cells to aloperine and positive olaparib (Figure 5C).

To gain further insight into the generalizability of the above results, wild-type or mutant IDH1/2-overexpressing HeLa cells and wild-type or mutant IDH1-isogenic MEF cells were used in our studies. IDH1/2-mutant HeLa cells were successfully identified (Figure S4A,B). In concordance with our findings in HCCC-9810 cells, IDH1/2-mutant HeLa cells showed higher sensitivity to aloperine and olaparib than IDH1/2 wild-type cells (Figure S4A,B). Similarly, treatment with aloperine significantly strengthened the growth inhibitory responses of IDH1-mutated MEF cells compared to the IDH1 wild-type cells (Figure S5). Thus, these data demonstrate that IDH1/2 mutation promotes cells’ sensitivity to aloperine, revealing that the levels of D-2HG determined the sensitivity of cells to aloperine.

### 2.5. Aloperine Reduced D-2HG Levels in RBE Cells by Decreasing Glutamate

**Figure 6:**
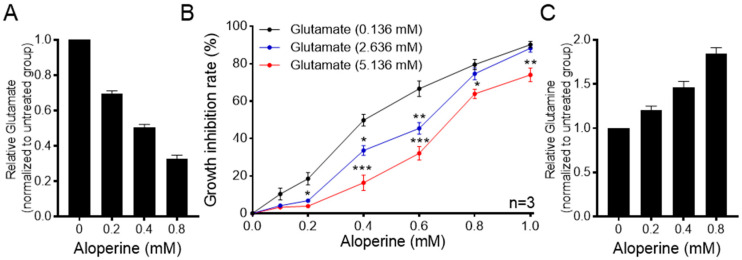
Aloperine preferentially inhibited RBE cells by decreasing glutamate. (**A**) Aloperine decreased the levels of glutamate in RBE cells. RBE cells were treated with increasing concentrations of aloperine for 24 h and subjected to LC–MS quantification analysis. The data are presented as the mean ± SEM (*n* = 3). (**B**) Exogenous glutamate supplementation reduced the antitumor activity of aloperine against RBE cells. RBE cells were co-treated with aloperine and different concentrations of glutamate for 72 h and subjected to in vitro antitumor assays. The data are presented as the mean ± SEM (*n* = 3). (**C**) Aloperine increased the levels of glutamine in RBE cells. RBE cells were treated with increasing concentrations of aloperine for 24 h and subjected to LC–MS quantification analysis. The data are presented as the mean ± SEM (*n* = 3). * *p* < 0.05, ** *p* < 0.01, *** *p* < 0.001.

According to the metabolomic results, glutamate content was significantly reduced in both RBE and HCCC-9810 cells by aloperine (Figure 3B). As previous studies have shown that glutamate is a major carbon source for the D-2HG in IDH-mutant cells [33], we proposed that a decrease in glutamate may be a factor in the reduction of D-2HG by aloperine in RBE cells. To address this issue, we first quantitatively evaluated the effect of aloperine on the levels of glutamate in RBE cells. Consistent with our metabolomic analysis-based findings, aloperine decreased levels of glutamate in a dose-dependent manner (Figure 6A). Then, we increased cellular glutamate levels in aloperine-treated RBE cells to determine whether this would abolish the antitumor activity of aloperine. Exposing RBE cells to increasing concentrations of glutamate significantly ameliorated the antitumor activity of aloperine (Figure 6B). We then addressed the mechanism via which glutamate reduced the inhibitory effect of aloperine on RBE cells. Cellular glutamate typically originates from two pathways: the glutaminase (GLS)-catalyzed hydrolysis of glutamine and the influx of extracellular glutamate by glutamate transporters [34]. Aloperine increased levels of glutamine in a dose-dependent manner (Figure 6B). In summary, aloperine decreased glutamate content by inhibiting the hydrolysis of glutamine.

### 2.6. Aloperine Preferentially Resensitizes RBE Cells to 5-Fluorouracil, AGI-5198 and Olaparib

**Figure 7:**
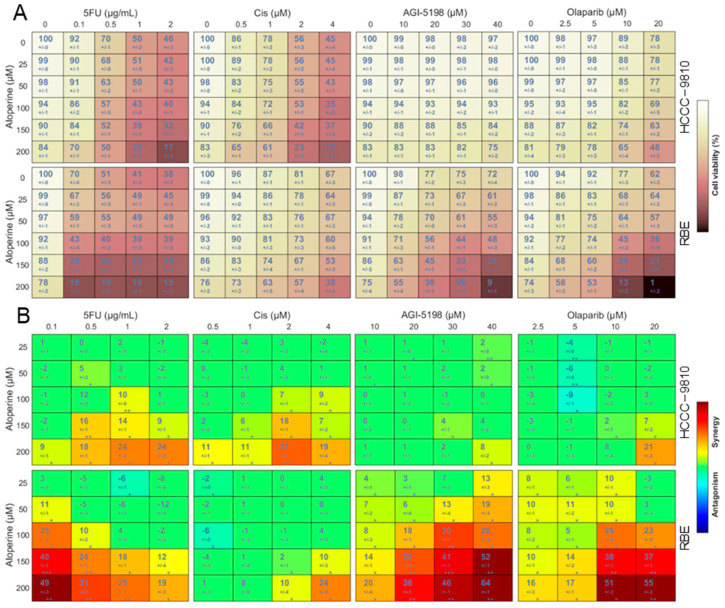
The synergetic/antagonistic effects of aloperine in combination with 5FU, Cis, AGI-5198, and AGI-120. (**A**) The cell viability of aloperine in combination with 5FU, Cis, AGI-5198, and AGI-120 in ICC cell lines. RBE and HCCC-9810 cells were treated with the indicated drug combination for 72 h and subjected to an in vitro antitumor assay. The data are presented as the mean ± SD (*n* = 3). (**B**) The synergy distribution of the ICC cells treated with the indicated drug combination. Cell viability results were analyzed using Combenefit software to obtain the synergy distribution. The data are synergy/antagonism scores for each combination and presented as the mean ± SD (*n* = 3). * *p* < 0.05, ** *p* < 0.01, *** *p* < 0.001.

To explore whether aloperine can be used as a chemotherapy-enhancing anti-ICC adjuvant drug, we evaluated the synergetic/antagonistic effect of aloperine in combination with four drugs: 5-fluorouracil (5FU), cisplatin (Cis), AGI-5198, and olaparib. HCCC-9810 and RBE cells were treated with aloperine (0–200 μM), 5FU (0–2 μg/mL), Cis (0–4 μM), AGI-5198 (0–40 μM), and olaparib (0–40 μM) via a combination treatment at a 1:1 ratio per dose (Figure 7A). The cells were treated with an increasing concentration of these drugs alone, and the drug’s cell-growth-inhibition rate did not exceed 50% (Figure 7A). Interestingly, compared with its use alone, the cell viability of aloperine in combination with 5FU, Cis, AGI-5198 and olaparib was significantly reduced at most concentrations, indicating that aloperine increased the anti-ICC activity of the tested chemotherapeutic drugs (Figure 7A).

Furthermore, to quantitatively evaluate the synergetic/antagonistic effect of each drug combination, drug combination-related cell viability data were analyzed using Combenefit 2.021 software via a classical Loewe synergy model. Fortuitously, aloperine + 5FU, aloperine + AGI-5198, and aloperine + olaparib combinations all displayed significant synergistic effects against RBE cells (Figure 7B). Conversely, aloperine + 5FU, aloperine + Cis, aloperine + AGI-5198, and aloperine + olaparib combinations all showed weak synergistic effects in HCCC-9810 cells, as did the aloperine + Cis combination in RBE cells (Figure 7B). In addition, some antagonistic drug combinations were observed at low-dose concentrations, such as the aloperine + 5FU and aloperine + Cis combinations in RBE cells (Figure 7B). In summary, aloperine preferentially resensitizes RBE cells to 5-fluorouracil, AGI-5198, and olaparib.

## 3. Discussion

Mutant IDH proteins acquire novel enzymatic activity to produce the oncometabolite D-2HG, which is thought to block cellular differentiation and promote the establishment of an immunosuppressive TME [35]. By comparing eight patients with an IDH1/2-mutated ICC to nine patients with a wild-type IDH1/2 ICC, we found significantly higher levels of 2HG in patients with IDH1/2 mutations versus the wild-type group [18]. Circulating 2HG may be a surrogate biomarker of IDH1 or IDH2 mutation status in ICC and correlate directly with tumor burden [36]. D-2HG is an inhibitor of many alpha-ketoglutarate (αKG)-dependent dioxygenases, leading to slow-accumulating alterations in the epigenetic landscapes of cells that result in oncogenic transformation [37]. D-2HG also promotes the establishment of an immunosuppressive tumor microenvironment. D-2HG could act as a direct inhibitor of lactate dehydrogenase in mouse T cells, altering glucose metabolism in T cells and inhibiting their proliferation, cytokine production levels, and ability to kill target cells [38]. Due to the above results, reducing the level of D-2HG has become a major strategy for the treatment of psoriasis. In this article, aloperine decreased the levels of D-2HG, and the levels of D-2HG were related to drug sensitivity, suggesting that D-2HG was involved in the antitumor activity of aloperine against IDH-mutant cells.

In IDH-mutant cells, the production of D-2HG was not only determined by the activity of mutant IDHs but also affected by the concentration of α-ketoglutarate [33,39]. Glutamate can be a primary α-ketoglutarate producer in IDH-mutant cells, whereas α-ketoglutarate is derived from the TCA cycle in wild-type IDH cells [33]. To achieve this, glutaminase is highly expressed in IDH-mutant cells compared to wild-type cells, leading to the increased conversion of glutamine to glutamate in IDH-mutant cells [40,41]. Thus, targeting glutaminase could improve the treatment of IDH-mutant malignancies [42]. In this study, aloperine decreased the levels of D-2HG and glutamate in a dose-dependent manner and increased levels of glutamines. These results support that aloperine decreased the levels of D-2HG by inhibiting the conversion of glutamine to glutamate, leading to the preferential cell growth inhibition of RBE cells compared to HCCC-9810 cells.

The mutation of IDH1 and IDH2 in ICC occurs in approximately 6.5–32% and 10% of cases, respectively [10]. Targeting the mutant IDH1/2 has opened a novel therapeutic avenue for ICC patients with IDH1 mutations, and the FDA have approved the mutant IDH1 inhibitor ivosidenib for treating IDH1-mutated advanced cholangiocarcinoma. The median survival rate for chemotherapy-refractory IDH1-mutant cholangiocarcinoma patients receiving ivosidenib therapy is 2.7 months, while that of patients treated with a placebo is 1.4 months [43]. Unfortunately, some studies have shown that acquired resistance to ivosidenib in ICC is increasing. The conversion of IDH1 R132C to IDH1 R132F and the acquired isoform mutation of IDH2 R172V have been reported in patients with IDH1-mutated cholangiocarcinoma who develop resistance to ivosidenib as a result [44,45]. To improve outcomes for patients with IDH-mutant tumors, treatment strategies other than directly inhibiting mutant IDH need to be explored [46]. In this study, aloperine decreased the levels of D-2HG by inhibiting the conversion of glutamine to glutamate via hydrolysis, overcoming the drug resistance induced by directly targeting mutant IDH.

In recent years, synergetic therapies have been very effective at treating IDH-mutant cancers [47]. At present, a variety of synergetic therapeutic strategies exist, the more successful ones of which are mIDH inhibitors in combination with chemotherapeutic agents, DNA damage inducers, and immune checkpoint inhibitors. For ICC, a phase 1b clinical trial (NCT03528642) was performed to study the side effects and optimal dose of the glutaminase inhibitor telaglenastat in combination with radiation therapy and temozolomide for treating patients with IDH-mutated astrocytoma [48]. Two phase 2 clinical trials were conducted to study the PARP inhibitor olaparib’s use in combination with durvalumab (NCT03991832) and ceralasertib (NCT03878095) to treat IDH-mutant cholangiocarcinoma [49,50]. Although these three clinical trials are at the recruitment stage, these synergetic therapies reveal a new trend in the treatment of IDH-mutant ICC. In this study, aloperine preferentially resensitizes RBE cells to 5-fluorouracil, AGI-5198, and olaparib, suggesting that aloperine could be developed as an adjuvant chemotherapy drug to treat ICC harboring mutant IDHs.

However, many limitations still need to be addressed. The anti-ICC activity assay of aloperine can only be carried out in cells, and further research is required for in vivo anti-ICC activity in mice. Based on our experimental results, we concluded that aloperine inhibits the conversion of glutamine to glutamate, which should be verified by an in vitro glutamine activity assay. Moreover, the underlying mechanism of aloperine showing ideal antitumor activity in IDH wild-type ICC cells should be addressed, as it could guide the obtainment of aloperine derivatives that better target IDH-mutant tumors.

## 4. Materials and Methods

### 4.1. Reagents

Matrine (Cat#: CSN19330), oxymatrine (Cat#: CSN19333), sophoridine (Cat#: CSN20630), sophocarpine (Cat#: CSN16469), cytisine (Cat#: CSN11123), and aloperine (Cat#: CSN10253) were acquired from CSNpharm (Shanghai, China). D-2-hydroxyglutarate (Cat#: T6820) was acquired from Targetmol (Shanghai, China). Octyl-D-2-hydroxyglutarate (Cat#: SML2200-5MG) was obtained from Sigma-Aldrich (Missouri, USA). Glutamate (Cat#: G0010) and glutamine (Cat#: G8230) were purchased from MedChemExpress (Shanghai, China). The anti-FLAG antibody (Cat#: 66008-4-Ig) was acquired from Proteintech (Wuhan, China) and the anti-GAPDH antibody (Cat#: ab181602) was acquired from Abcam (Cambridge, England). The MimiColor Protein Marker (Cat#: M1023) was acquired from Minibio Technology Co., Ltd. (Taiyuan, China). DMEM medium (Cat#: 01-052-1ACS) and RPMI-1640 medium (Cat#: 01-100-1ACS) were purchased from Biological Industries (Kibbutz Beit-Haemek, Israel). Fetal bovine serum (FBS) (Cat#: S660JJ) was acquired from Shanghai BasalMedia Technology Co., Ltd. (Shanghai, China).

### 4.2. Cell Culture

RBE, HCCC-9810, 293T/17, and HeLa cells were sourced from the Cell Bank of the Shanghai Institute for Biological Sciences, the Chinese Academy of Science. MEF cells harboring wild-type IDH1 or IDH1 R132Q were obtained from Professor Li Qinxi of the School of Life Sciences, Xiamen University (Xiamen, Fujian, China) [51]. 293T/17, HeLa, and MEF cells were cultured in DMEM complete medium, while RBE and HCCC-9810 cells were cultured in RPMI-1640 complete medium. All complete media included 10% FBS, 100 μg/mL of penicillin, and 100 μg/mL of streptomycin. All cells were incubated in a 5% CO_2_ humidified atmosphere at 37 °C.

### 4.3. Plasmid Construction

The lentiviral expression vector pRSV-CMV-Flag-EF1-Puro-T2A-GFP was obtained in the manner previously described [52]. The cDNAs of wild-type and mutant IDH1/2 were obtained from Professor Li Qinxi of the School of Life Sciences, Xiamen University (Fujian, China) and sub-cloned into the BamH I and Xba I sites of pRSV-CMV-Flag-EF1-Puro-T2A-GFP using the Exo III-assisted ligase-independent cloning method [11].

### 4.4. Lentivirus Infection and Stable Cell-Line Generation

The lentivirus was prepared by co-transfecting a recombinant lentiviral expression vector with psPAX2 and pMD2.G (2:2:1) into 293T/17 cells using PEI 40000 (YEASEN, Cat#: 40816ES02, Shanghai, China) according to the manufacturer’s instructions. The lentivirus-containing supernatant was collected 72 h post-transfection, filtered, and mixed with an equal volume of fresh media containing 10 μg/mL of polybrene to prepare the infected mixture. For the lentivirus infection, the target cells were incubated with the infected mixture for 24 h. For stable cell-line generation, infected cells were selected using puromycin (5 μg/mL) for 2 weeks to generate stably expressing cells.

### 4.5. In Vitro Antitumor Assay

The in vitro antitumor assay was performed using the SRB assay. Cells were seeded in 96-well plates at a density of 3000–5000 cells per well according to cell type and incubated for 24 h. After attachment to the plate, cells were exposed to drugs for 72 h. Then, upon removing the cell culture medium, cells were fixed with 100 μL of 10% trichloroacetic acid at 4 °C for more than 2 h. The plate was washed four times with slow-running tap water and completely dried at room temperature. Cells were stained with 0.4% SRB solution for 20 min and then rinsed with 1% acetic acid five times. The cell-binding dye was dissolved in 10 mM of Tris solution. Afterward, the optical density (OD) values were measured at 510 nm using a Microplate Reader (Tecan). The cell growth inhibition rate (%) was calculated using (OD_control_ − OD_group_)/(OD_control_ − OD_blank_) × 100, where OD_control_ is the OD value in the presence of 0.1% DMSO, OD_group_ is the OD value in the presence of drugs, and OD_blank_ is the OD value in the absence of cells.

### 4.6. Synergy and Antagonism Analysis

Synergy and antagonism analysis of drug combinations was performed using Combenefit software (https://sourceforge.net/projects/combenefit/ (8 July 2024)). HCCC-9810 and RBE cells were treated with one drug alone or two drugs in combination for 72 h before being subjected to an SRB assay to obtain the in vitro antitumor activity of drug treatment. These data were analyzed using Combenefit software via the Loewe model.

### 4.7. Preparation of Cellular Metabolites

Cells were treated with aloperine for 24 h. After washing with cold PBS, the collected cells were quenched using a methanol/water mixture (80:20) at −80 °C. Quenched cells were collected using a cell scraper and centrifuged at 14,000× *g* for 15 min at 4 °C to obtain the supernatant. The supernatant was dried using Concentrator plus (Eppendorf) and dissolved in acetonitrile with 0.1% formic acid. This dissolved mixture was centrifuged at 14,000× *g* for 15 min, and the supernatant was collected in a new centrifuge tube to obtain cellular metabolites.

### 4.8. Metabolomics Analysis and Data Processing and Analysis

Cellular metabolite samples underwent LC–MS-based metabolomics analysis using a Thermo-Fisher Dionex UltiMate 3000 UHPLC-Q Exactive Orbitrap-MS/MS system. The samples were subjected to chromatographic separation via an Acquity UPLC HSS T3 column. The chromatographic separation conditions and mass spectrometry analysis parameters referred to in [53] were used. The ion intensities of each peak were obtained from the LC–MS raw data using Compound Discoverer 3.0 software and normalized via total ion density in Microsoft Excel 365. The data of normalized ion intensities were analyzed using SIMCA-P 14.1 software (Umetrics, Sweden) via principal component analysis (PCA), and orthogonal partial least squares-discriminant analysis (OPLS-DA). The regression lines of R2 and Q2 intersected on the right, meaning that the model of OPLS-DA analysis was accurate. Differential metabolites were screened via VIP values (VIP >1) and *t*-tests (*p* < 0.05) in the S-plot. Moreover, Compound Discoverer 3.0 software was used to identify the compound names of ion peaks.

### 4.9. Quantification Analysis of D-2HG, Glutamate and Glutamine

Cellular metabolites were prepared and analyzed via the methods described in Section 4.7 and Section 4.8. LC–MS analysis was performed to quantitatively measure the levels of D-2HG, glutamate, and glutamine by comparing peak areas with pure metabolite standards. The total protein levels used for the subsequent normalization of the LC–MS ion intensities were determined from equivalently treated control plates to obtain the normalized peak area. The relative levels of glutamate and glutamine were calculated as follows: normalized peak area (treated concentration)/normalized peak area (untreated).

### 4.10. Western Blotting

Cells were harvested using a cell scraper and directly sampled using 1 × loading buffer. The proteins of samples were separated via SDS-PAGE and transferred to a PVDF membrane (Millipore, MA, USA). The membrane was incubated with a 5% Bovine Serum Albumin solution formulated in TBST (50 mM Tris-HCl, 150 mM NaCl, 0.05% Tween 20, pH 7.6) for 1 h at room temperature. Following incubation overnight with specific primary antibodies at 4 °C, the membrane was washed with TBST at room temperature. Then, the membrane was incubated with horseradish peroxidase-conjugated secondary antibodies for 1 h. Chemiluminescence was detected using an enhanced chemiluminescence reagent on a ChemiDoc XRS+ imaging system (Bio-Rad, Hercules, CA, USA).

### 4.11. Statistical Analysis

Data analyses were performed using GraphPad Prism 8.0. Significant differences between all pairwise combinations were determined using a two-way ANOVA multiple comparison test; a one-way ANOVA was applied to unpaired comparisons with one treatment. Values of *p* < 0.05 were considered significant. Significance levels are indicated by * *p* < 0.05, ** *p* < 0.01, *** *p* < 0.001, and **** *p* < 0.0001; ns indicates ‘not significant’.

## 5. Conclusions

**Figure 8:**
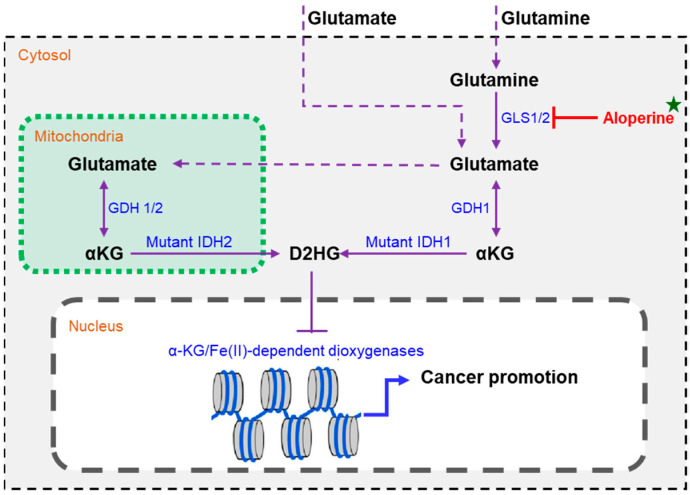
The proposed mechanism through which aloperine preferentially inhibits the growth of intrahepatic cholangiocarcinoma cells harboring the mutant IDH1.

In conclusion, the in vitro anti-ICC activities of six quinolizidine alkaloids were explored. Aloperine was the most potent antitumor compound among the tested quinolizidine alkaloids with selective inhibitory effects on RBE and HCCC-9810 cells. The IDH1/2 mutation promotes cells’ sensitivity to aloperine. Aloperine decreased the levels of D-2HG and glutamate in a dose-dependent manner and increased levels of glutamine. Exogenous D-2HG and glutamate supplementation reduced the antitumor activity of aloperine against RBE cells. In conclusion, these results suggest that aloperine decreased glutamate content by inhibiting the hydrolysis of glutamine, potentially reducing D-2HG levels and, consequently, leading to preferential growth inhibition in IDH-mutant cells (Figure 8). In addition, aloperine preferentially resensitized RBE cells to 5-fluorouracil, AGI-5198, and olaparib. This study found that aloperine could be developed as a lead drug or adjuvant chemotherapy drug to treat cancer harboring mutant IDHs, which is crucial for ICC drug development.

## Data Availability

All data generated or analysed during this study are included in this published article.

## Supplementary Materials

The following supporting information can be downloaded at: https://www.mdpi.com/article/10.3390/ijms25179226/s1.

## Abbreviations

ICC: Intrahepatic cholangiocarcinoma; IDH: isocitrate dehydrogenase; FGFR: fibroblast growth factor receptor; D-2HG: D-2-hydroxyglutarate; LC–MS: liquid chromatography–mass spectrometry; GLS: glutaminase; 5FU: 5-fluorouracil; Cis: cisplatin; αKG: alpha-ketoglutarate; TCA: tricarboxylic acid cycle; SEM: standard error of the mean; IC_50_: half-maximal inhibitory concentration.
