# Supplementary material for: Discovery of Aloperine as a Potential Antineoplastic Agent for Cholangiocarcinoma Harboring Mutant IDH1
Source: Int J Mol Sci. 2024 Aug 25;25(17):9226. doi: 10.3390/ijms25179226 (PMC11395030; doi:10.3390/ijms25179226)
Supplement: Supplementary file 1 [file ijms-25-09226-s001.zip › ijms-3121772-supplementary.pdf]

Discovery of aloperine as a potential antineoplastic agent for cholangiocarcinoma harboring mutant IDH1

supplementary materials

Xingkang Wu[1,*], Yang Li[1], Chenchen Han[1], Shifei Li[2], Xuemei Qin[1,*]

[1] Modern Research Center for Traditional Chinese Medicine, the Key Laboratory of Chemical Biology and Molecular Engineering of Ministry of Education, Shanxi University, No. 92, Wucheng Road, Taiyuan 030006, China.

[2] Key Laboratory of Chemical Biology and Molecular Engineering of Education Ministry, Institute of Molecular Science, Shanxi University, No. 92, Wucheng Road, Taiyuan 030006, China.

[*]**Corresponding author:** Xingkang Wu, wuxingkang@sxu.edu.cn; Xuemei Qin, qinxm@sxu.edu.cn

E-mail addresses: Xingkang Wu, wuxingkang@sxu.edu.cn; Yang Li, ly20171310418@163.com; Chenchen Han, 15735649396@163.com; Shifei Li, lisf@sxu.edu.cn; Xuemei Qin, qinxm@sxu.edu.cn

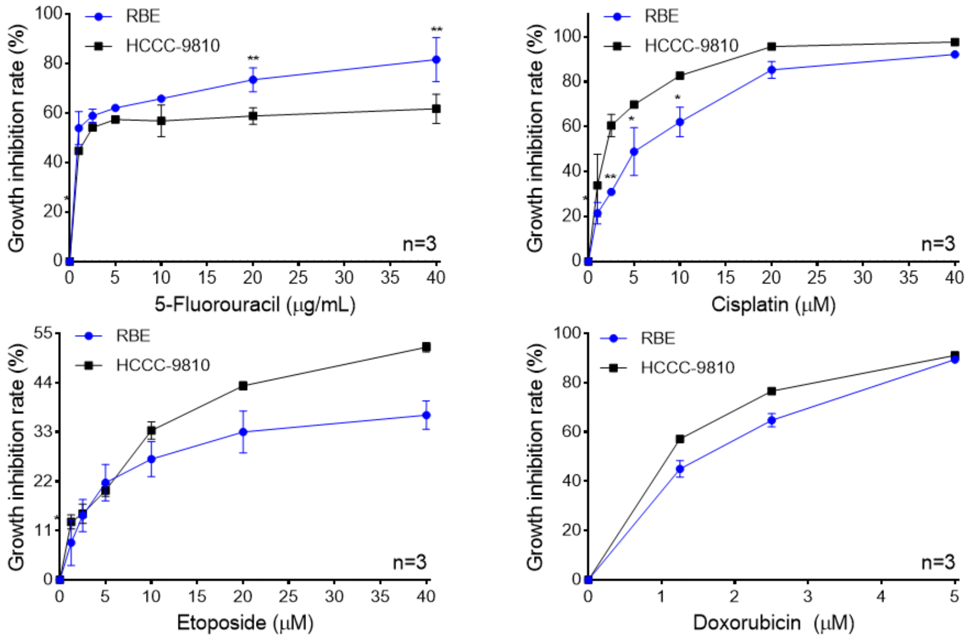

Figure S1. The growth inhibitory activities of four commonly used anti-tumor drugs against ICC cells.

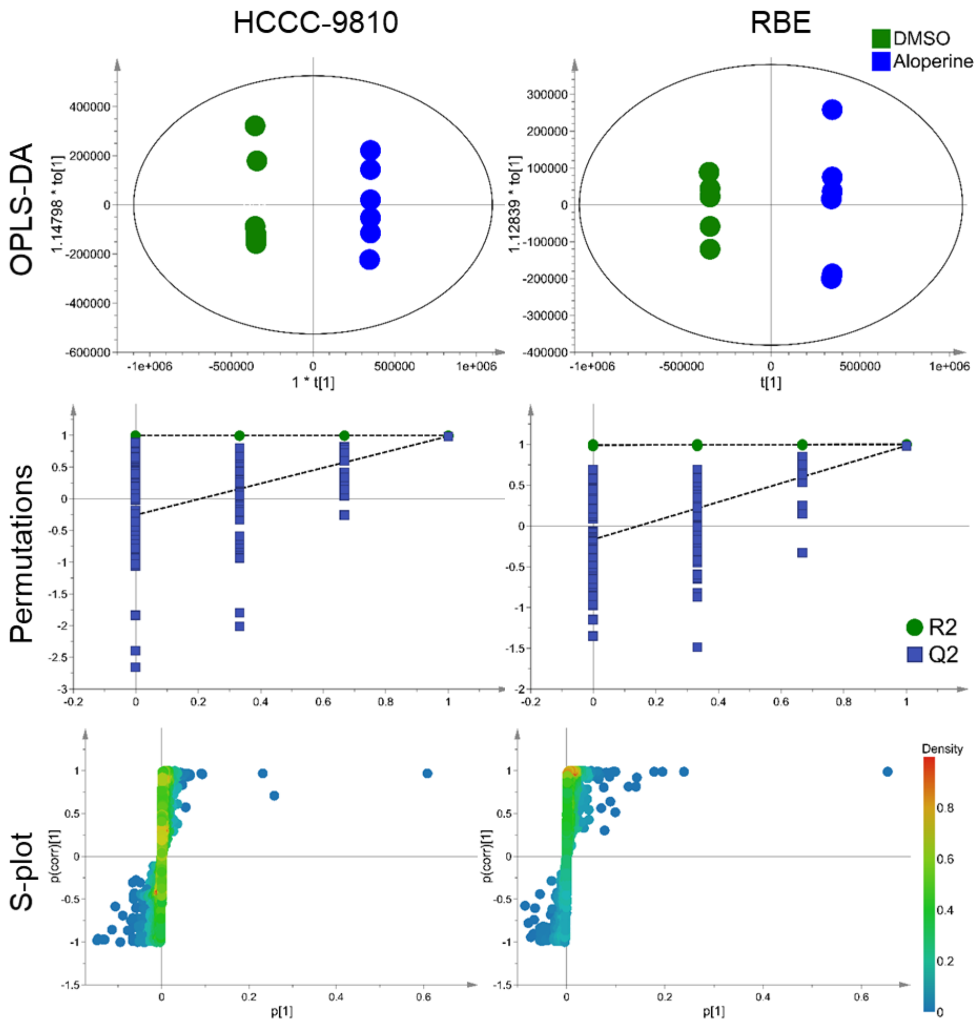

Figure S2. The OPLS-DA analysis of metabolites sampled from aloperine-treated and -untreated

ICC cells.

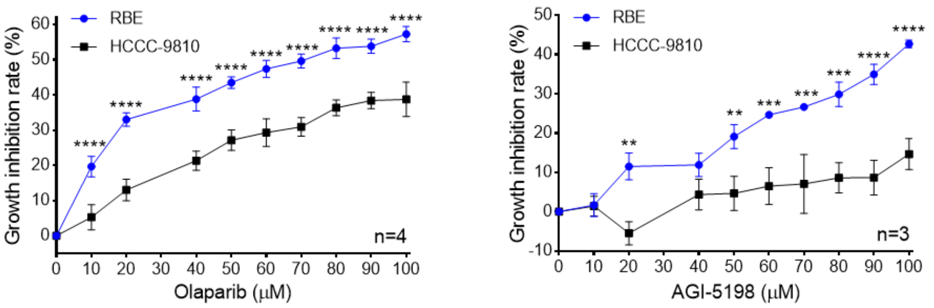

Figure S3. The cell growth inhibitory activities of two IDH-mutant cells targeting agents against ICC cells.

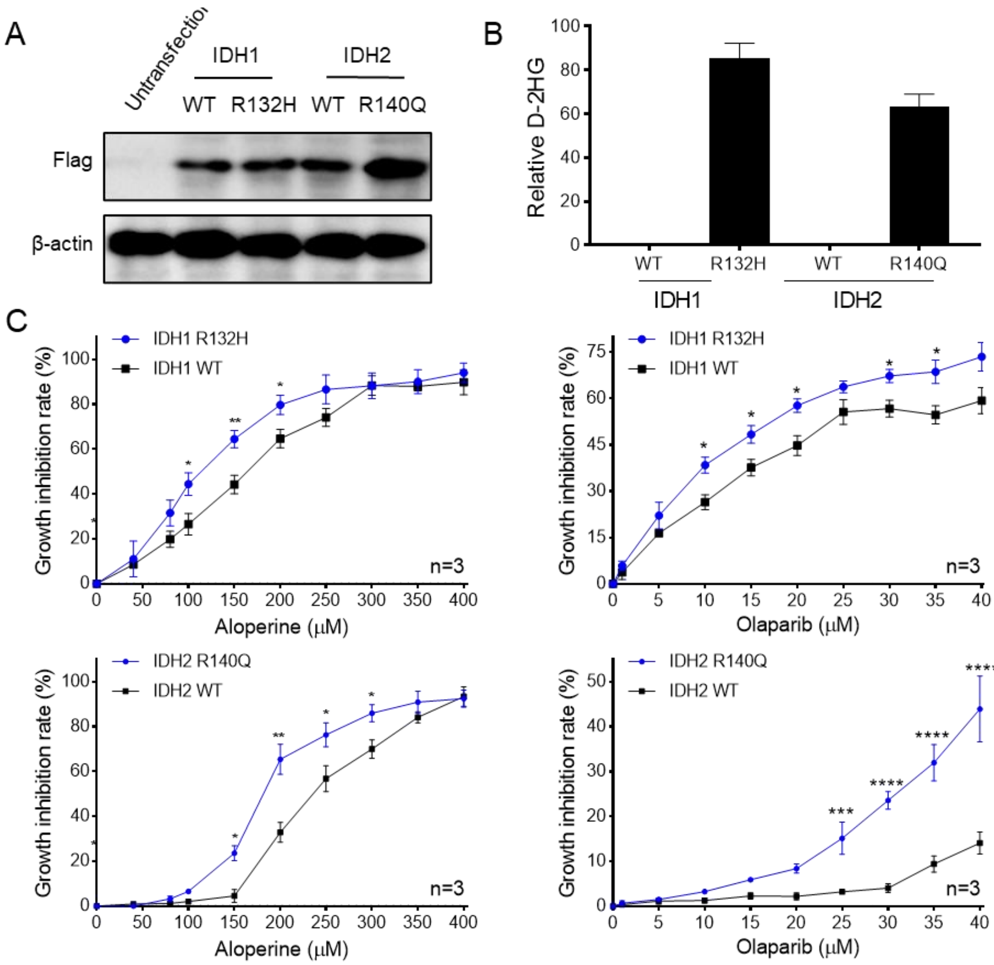

Figure S4. IDH1/2 mutation sensitizes HeLa cells to aloperine. (A) The protein expression of transfected IDH1/2 in HeLa cells. HeLa cells, stably expressing wild type or mutant IDH1/2, were subjected to western blotting. (B) The D-2HG levels of transfected IDH1/2 in HeLa cells. LC-MS analysis was performed to measure the levels of cellular D-2HG by using metabolite standards. The data were presented as the mean ± SEM (n = 3). (C) The cell growth inhibitory activities of aloperine against HeLa cells with wild type or mutant IDH1/2. HeLa cells, stably expressing wild type or mutant IDH1/2, were treated by indicated concentrations of indicated aloperine for 72 h, and subjected to in vitro antitumor assays. The data were presented as the mean ± SEM (n = 3).

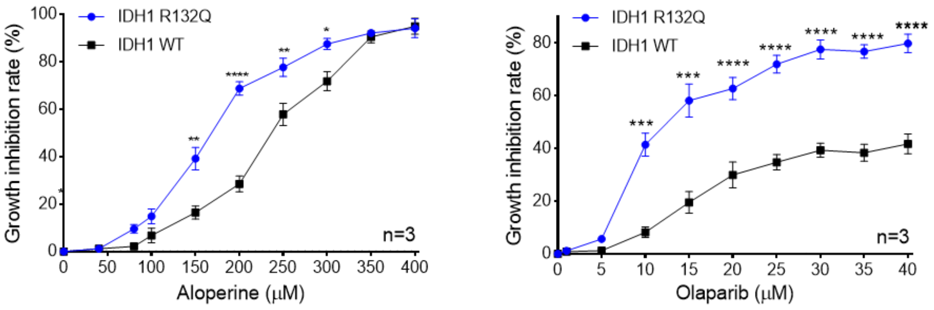

Figure S5. IDH1/2 mutation sensitizes MEF cells to aloperine. MEF cells harboring wild type or R132Q mutant IDH1, were treated by indicated concentrations of indicated aloperine for 72 h, and subjected to in vitro antitumor assays. The data were presented as the mean ± SEM (n = 3).
